# Diagnostic accuracy of artificial intelligence-aided capsule endoscopy (TOP100) in overt small bowel bleeding

**DOI:** 10.1007/s00464-023-10273-w

**Published:** 2023-07-26

**Authors:** Antonio Giordano, Miriam Escapa, Miquel Urpí-Ferreruela, Gherzon Casanova, Gloria Fernández-Esparrach, Àngels Ginès, Josep Llach, Begoña González-Suárez

**Affiliations:** 1 Endoscopy Unit, Gastroenterology Department, ICMDM, Hospital Clínic de Barcelona, Barcelona, Spain; 2 Institut d’Investigacions Biomèdiques August Pi i Sunyer (IDIBAPS), Barcelona, Spain; 3 Centro de Investigación Biomédica en Red de Enfermedades Hepáticas y Digestivas (CIBEREHD), Barcelona, Spain; 4 Faculty of Medicine, University of Barcelona, Barcelona, Spain

**Keywords:** Artificial intelligence, Hemorrhage, Capsule endoscopy, Intestine, Small

## Abstract

**Background:**

Capsule endoscopy (CE) is the first-choice exploration in case of overt small bowel bleeding (SBB). An early CE is known to increase diagnostic yield, but long reading times may delay therapeutics. The study evaluates the diagnostic performance of the artificial intelligence tool TOP100 in patients with overt SBB undergoing early CE with Pillcam SB3.

**Methods:**

Patients who underwent early CE (up to 14 days from the bleeding episode) for suspected overt SBB were included. One experienced endoscopist prospectively performed standard reading (SR) and a second blind experienced endoscopist performed a TOP100-based reading (TR). The primary endpoint was TR diagnostic accuracy for lesions with high bleeding potential (P2).

**Results:**

A total of 111 patients were analyzed. The most common clinical presentation was melena (64%). CE showed angiodysplasias in 40.5% of patients (45/111). In per-patient analysis, TR showed a sensitivity of 90.48% (95% CI 82.09–95.80), specificity of 100% (95% CI 87.23–100) with a PPV of 100% (95% CI 94.01–100), NPV of 77.14% (95% CI 63.58–86.71) and diagnostic accuracy of 92.79 (86.29–96.84). At multivariate analysis, adequate intestinal cleansing was the only independent predictor of concordance between TR and SR (OR 2.909, *p* = 0.019). The median reading time for SR and TR was 23 min (18.0–26.8) and 1.9 min (range 1.7–2.1), respectively (*p* < 0.001).

**Conclusions:**

TOP100 provides a fast-reading mode for early CE in case of overt small bowel bleeding. It identifies most patients with active bleeding and angiodysplasias, aiding in the prioritization of therapeutic procedures. However, its accuracy in detecting ulcers, varices and P1 lesions seems insufficient.

**Graphical abstract:**

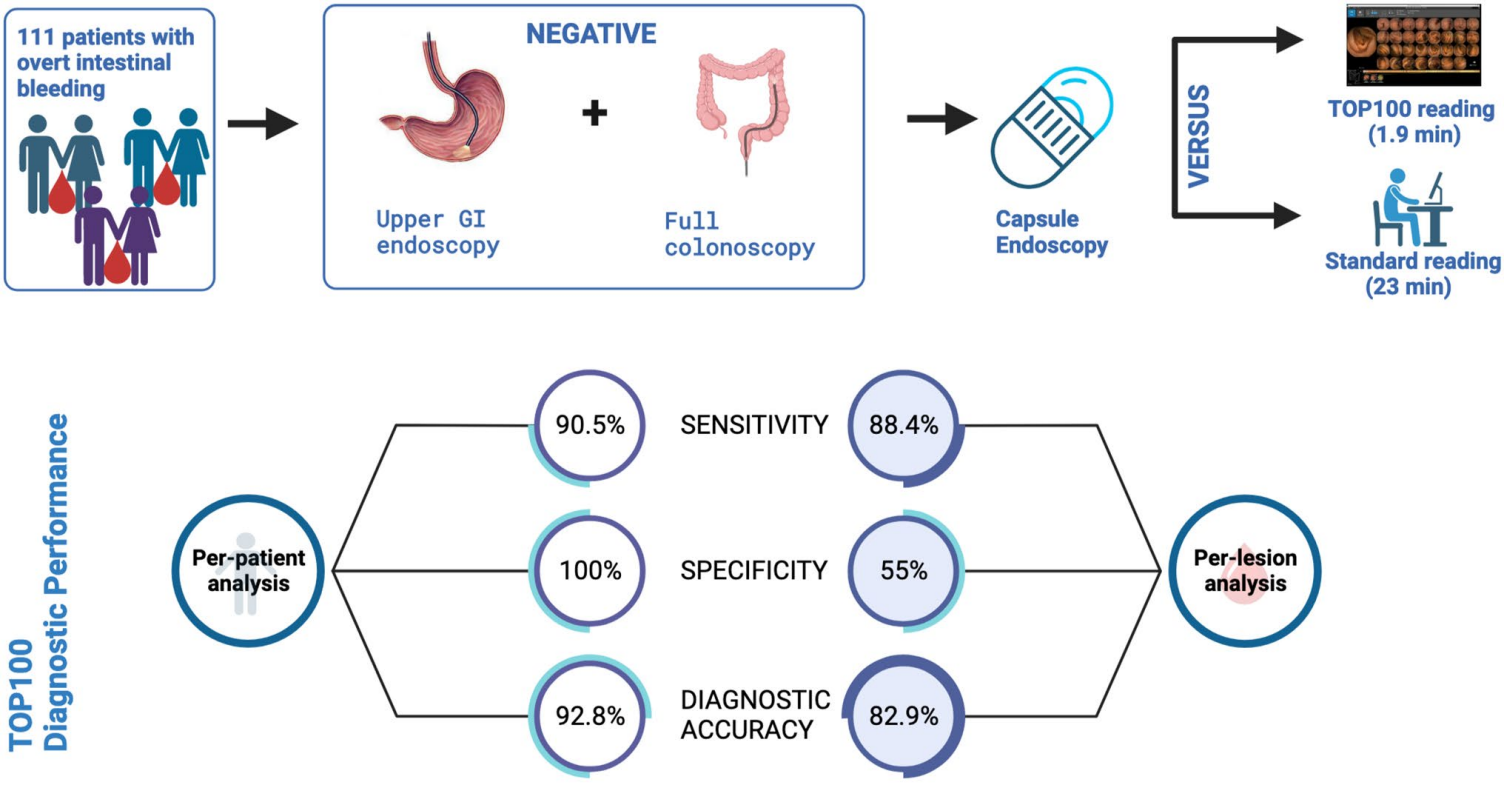

Small bowel bleeding (SBB) accounts for 5 to 10% of all gastrointestinal bleeding and is defined as overt or occult bleeding which originates in the small bowel (SB) in patients with an inconclusive exploration of the upper and lower gastrointestinal tracts (formerly named obscure gastrointestinal bleeding). [1, 2] It represents a cause of hospital admission and blood transfusion, resulting in a mortality rate of up to 10% [3]. Major guidelines in Europe and the United States address small bowel capsule endoscopy (SBCE) as the mainstay technique to explore the SB and detect the cause of bleeding [4, 5]. Alternative techniques may be represented by CT or Magnetic Resonance Imaging enterography, especially in the case of solid or extramural lesions. On the other hand, balloon and spiral enteroscopy, which represent more invasive techniques, are limited to therapeutic use or to a “diagnose and treat” strategy in the case of early (< 72 h) overt bleeding [5].

Nowadays, SBCE represents a safe and efficient technique. [6] However, due to long reading times, the procedure may be exhausting and efficiency depends on reader experience and proper reading technique. [7] Recently, the introduction of artificial intelligence (AI) has spread in all fields of medicine. AI may help identify or diagnose SB lesions, thus reducing reading times [8].

Pillcam SB3 (Medtronic, Minneapolis, USA) has been the first capsule endoscopy device to integrate AI tools in its reading software (Pillcam™ Reader, previously Rapid Reader, Medtronic, USA) to assist with video assessment and potentially reduce reading times [9]. TOP100 is an integrated AI tool that selects the 100 most relevant frames, including potential lesions from SBCE video recording. In a study by Arieira et al. evaluating patients with SBB, mainly represented by occult presentation (90.7%), TOP100 was able to identify 83.5% of significant lesions [10]. However, these values are insufficient to completely rely on TOP100 reading for SBCE assessment, especially in the case of occult SBB which requires an accurate evaluation for the indication of further studies or therapeutic procedures.

Little is known about the utility and impact of TOP100 on lesion detection and reading times in acute and urgent settings like overt SBB, where a rapid diagnosis is needed to schedule the appropriate therapeutic procedure. The present study aimed to evaluate the diagnostic performance of TOP100 in patients submitted to early SBCE due to overt SBB.

## Materials and methods

### Study design

Research was conducted in the SBCE database of Hospital Clínic of Barcelona, searching for all consecutive early SBCE explorations performed with the Pillcam SB3 Capsule Endoscopy from March 2018 to March 2021. Inclusion criteria were: (1) SBCE indication for overt suspected small bowel bleeding (intestinal bleeding with negative upper gastrointestinal endoscopy and ileo-colonoscopy, formerly obscure gastrointestinal bleeding); (2) recent bleeding (up to 14 days from SBCE placement); (3) availability of TOP100 tool in reading software. Exclusion criteria were: (1) incomplete capsule studies; (2) patients with no upper and lower gastrointestinal endoscopy before undergoing SBCE; (3) repeated SBCE explorations in the same patient.

A new reading was performed for all SBCE videos. The study’s primary endpoint was to assess TOP100 diagnostic performance (sensitivity, specificity, positive predictive value, negative predictive value, accuracy) for P2 lesions in patients with overt SBB in comparison with standard reading (SR) as the gold standard. Secondary endpoints were: (1) to compare SBCE reading time between SR and TOP100 (TR); (2) to assess the diagnostic performance of TOP100 for P1 lesions; (3) to detect predictors of concordance between TR and SR.

The study was conducted in accordance with the Declaration of Helsinki and approved by the Institutional Ethics Committee (IEC) of Hospital Clínic de Barcelona (HCB/2020/1204). Informed consent was waived due to the study design and the use of anonymized data according to IEC requirements and local laws.

### Capsule endoscopy protocol

A Pillcam SB3® (Medtronic, Minnesota, USA) was used in all patients. A 12-h clear liquid diet and 8-h fasting from solid food were requested before the procedure. A 1L polyethyleneglycol bowel preparation (Moviprep, Norgine Pharmaceuticals) had to be ingested 2 h before the procedure. Prokinetic drug (Metoclopramide) was administered 15 min before capsule ingestion in all inpatients according to local protocols, except for drug contraindication. The endoscopic capsule was swallowed with 200 ml of water with 300 mg of simethicone. Patients could start a liquid diet at 2 h from capsule ingestion followed by a normal solid diet after 4 h.

### Capsule exploration assessment

**Fig. 1:**
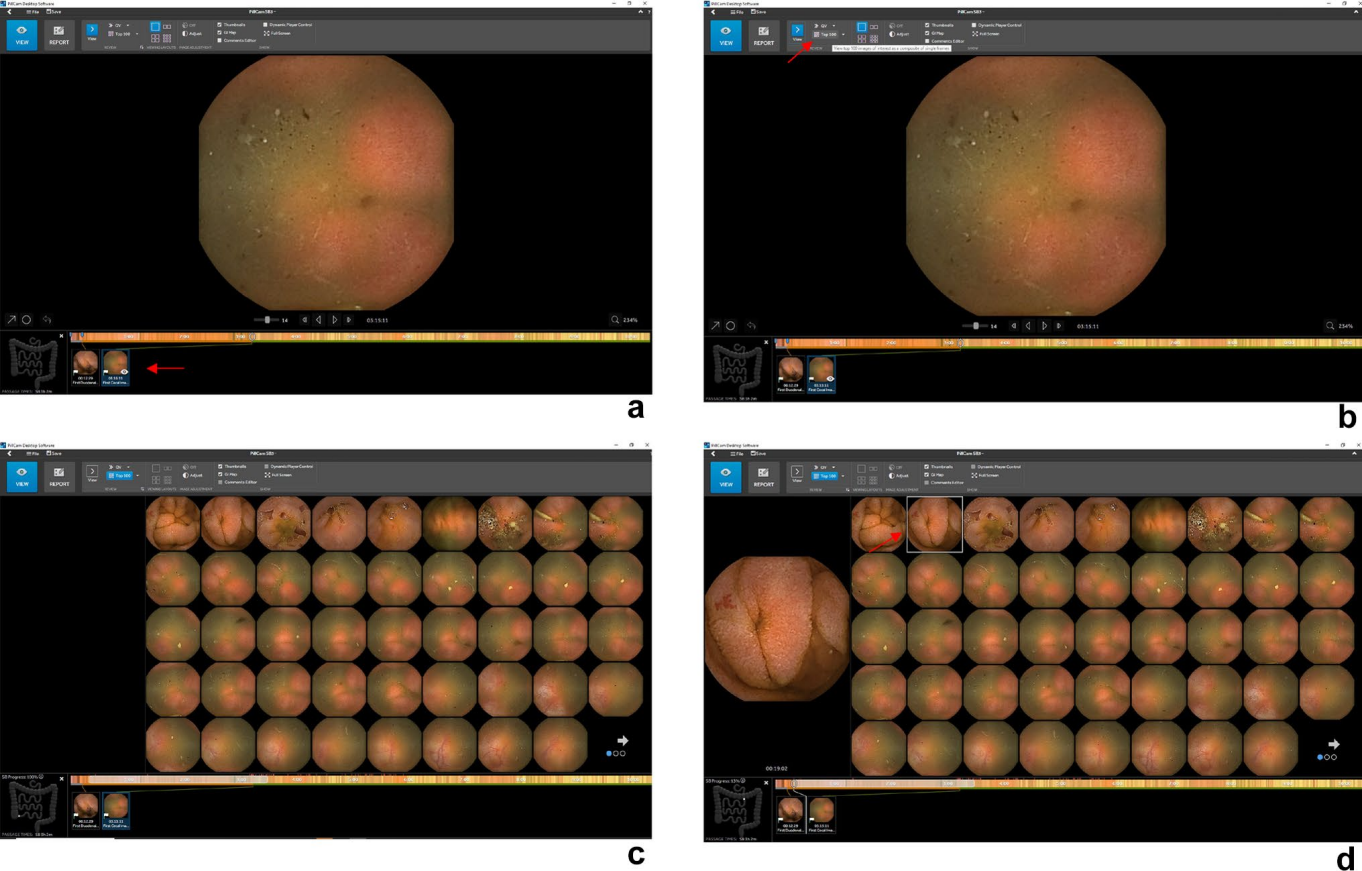
Application of the TOP100 Tool in Pillcam™ Reader Software v9. The TOP100 Tool, an integrated AI feature within the Pillcam™ Reader Software v9, enhances the reading process. To begin a TOP100-based reading, the user is required to identify and select the initial duodenal and cecal images (**a**). By clicking on the TOP100 icon, the tool is activated (**b**). The TOP100 Tool automatically identifies and displays 100 frames that may contain significant findings (**c**). The user has to review each frame and choose to accept or reject them (**d**)

SBCE procedures included in the analysis were first anonymized and coded. Two independent endoscopists (AG & BGS), with experience in more than 1000 endoscopic capsule explorations, performed the new SBCE reading. Both endoscopists were blind to the previous report on the exploration, patients’ demographic, and clinical background. One endoscopist performed SR according to ESGE (European Society of Gastrointestinal Endoscopy) guidelines [11]: briefly, the anonymized capsule video was marked at the first gastric, first duodenal, and first cecal image and read using Pillcam™ Reader Software at a speed of no more than 10 frames per second. The assessment was limited to the SB (first duodenal image to first cecal image). All detected lesions were registered on a coded digital sheet according to type, number, and location. Reading time was measured with a digital timer and included the marking of the first duodenal and cecal image, SB reading, and lesion identification, whereas the image review and report writing were excluded. A second endoscopist performed the reading based on TOP100 images (TR). The video was marked as for SR and by the activation of the TOP100 tool, a full screen reading of the static frames presented by the software was carried out **(**Fig. 1**)**. Lesions characterization and reading time were assessed as per SR.

### Lesion identification and characterization

All detected lesions were classified as per Saurin classification [12, 13]: P0 lesions (with no potential for bleeding: submucosal veins, diverticula without the presence of blood or nodules without mucosal breaks), P1 lesions (with uncertain hemorrhagic potential: red spots, small or isolated erosions without bleeding), and P2 lesions (with high potential for bleeding: typical angiodysplasias, multiple erosions areas, ulcers, active bleeding/visible blood, tumors, and varices). Only P2 and P1 lesions were considered for the analysis. In the case of the detection of multiple identical lesions, the differentiation was performed according to three main characteristics: the aspect of the lesion, the surrounding mucosa, and the time interval between lesions.

### Study variables definition

Diagnostic yield was defined as the number (percentage) of patients with at least one P2 lesion. The detection rate was defined as the percentage of patients or lesions found by TR in comparison with SR; lesions not confirmed by SR were considered false positives.

SB cleansing was assessed according to a qualitative scale (excellent, good, fair, poor) described by Brotz et al. [14]. Excellent and good cleansing was considered adequate intestinal preparation.

### Statistics

Data were analyzed with the Kolmogorov–Smirnov test to exclude normal distribution. Qualitative variables were expressed as absolute numbers and percentages, quantitative values were expressed as median and interquartile range (IQR). Positive predictive value (PPV), negative predictive value (NPV), and diagnostic accuracy were expressed as percentages and 95% confidence interval (95% CI). Reading time was measured in minutes and seconds and expressed in decimals. The concordance between SR and TR was calculated with Cohen’s kappa coefficient (per-lesion analysis). Univariate analysis was conducted with the Mann–Whitney *U* test or Chi-squared test, when appropriate. Predictors of concordance were entered in a multivariate model (forward stepwise method) and analyzed with multiple binary logistic regression. Statistical analysis was performed with SPSS Statistics for Windows, Version 26.0 (IBM Corp. Released 2019. Armonk, NY, USA).

## Results

### Study population

**Fig. 2:**
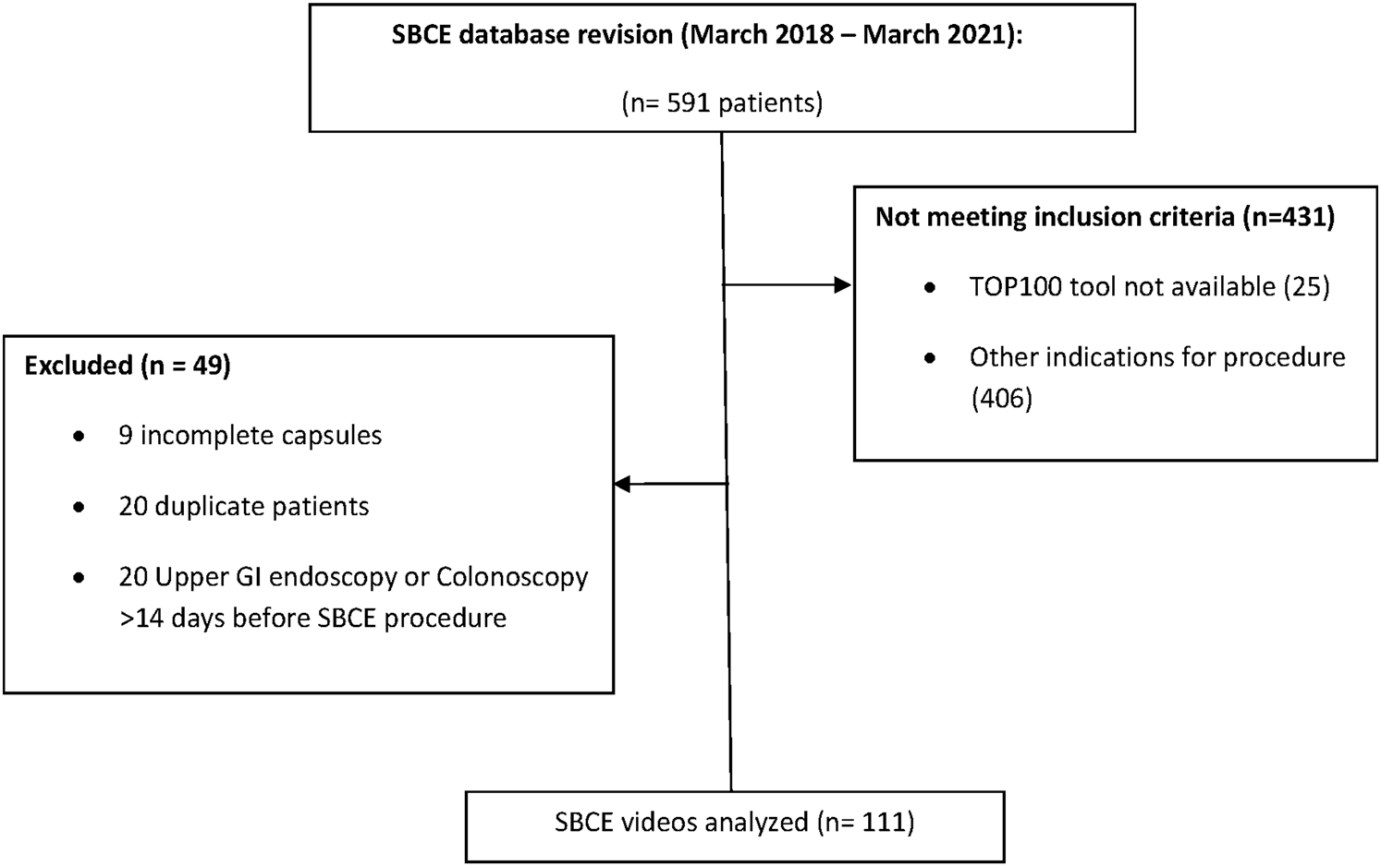
Flow-chart of inclusion and exclusion criteria

From March 2018 to March 2021 a total of 591 SBCE videos were found. Of these, 431 were excluded for not meeting the inclusion criteria, and 49 procedures for being incomplete explorations (*n* = 9), duplicate patients (*n* = 20), or having a prior upper gastrointestinal endoscopy or ileo-colonoscopy realized > 14 days before SBCE (*n* = 20). Finally, 111 SBCE videos were analyzed (Fig. 2). The patient population presented a median age of 69 years (56–78), with 63.1% of males; at the time of SBCE, 74.8% were inpatients. In 48.6% of patients (54/111), bleeding was active in the last 24 h before SBCE exploration. The most common presentation for SBB was melena (64%). Globally, 59.5% (64/109) of patients were transfused due to SBB. Anticoagulants (34.2%) and anti-platelet drugs (9.9%) were the most common concomitant medications with bleeding risk. Demographic characteristics are extensively described in Table 1.

**Table 1:** Demographic characteristics of the study population

*N*	111	
Age, yrs (IQR)	69	56–78
Males, *n* (%)	70	63.1%
Inpatients, *n* (%)	83	74.8%
Active bleeding (< 24 h), *n* (%)	54	48.6%
Days from last bleeding, median (IQR)	2	1–8
Days from first bleeding, median (IQR)	6	3–12
Hb, g/L (IQR)	82	67–101.5
Transfusion requirement	66	59.5
Type of bleeding, *n* (%)		
Melena	71	64.0
Haematoquezia	40	36.0
Comorbidities, *n* (%)		
Cardiovascular disease	98	88.3
Diabetes	22	19.8
Chronic liver disease	20	18.0
Chronic kidney disease	27	24.3
Bleeding risk medications, *n* (%)		
Anticoagulants	38	34.2%
Antiplatelet	11	9.9%
NSAIDs	3	2.7%
None	59	53.2%

*IQR* interquartile range; *NSAID* non-steroidal anti-inflammatory drug

### Capsule endoscopy overall performance

Complete technical data related to SBCE explorations are presented in Table 2. The use of prokinetics (metoclopramide) was adopted in 72.1% of patients and SB cleansing was adequate in 83.8% of patients (93/111). The median reading times of SR and TR were statistically different (*p* < 0.001), accounting for 23 min (18.0–26.8) and 1.9 min (range 1.7–2.1), respectively. Diagnostic yield resulted in 75.7% (84/111) for SR and 68.5% (76/111) for TR. The diagnostic yield for P1 lesions was 29.7% (33/111) for SR and 22.5% (25/111) for TR, with a significant concordance coefficient of 0.74.

**Table 2:** Technical data of SBCE exploration

GTT, minutes (IQR)	14	(7–34)
ITT, minutes (IQR)	289	(199–357)
Adequate cleansing, *n* (%)	93	(83.8%)
Metoclopramide, *n* (%)	80	(72.1%)

*GTT* gastric transit time; *ITT* intestinal transit time

### Detection performance of TOP100—per-patient analysis

TR showed an overall detection rate of 90.5% (76/84 patients), with 100% in case of active bleeding (12/12), 97.7% for angiodysplasias (44/45), 76.5% for ulcers (13/17), 75% for multiple erosions areas (3/4) and 66.7% for varices (4/6).

Overall, TOP100 demonstrated an accuracy of 92.79% for the identification of patients with P2 lesions, with high sensitivity (90.48%) and specificity (100%). As regards P1 lesions, SR identified 34 positive patients, of these 25 were detected by TR with a sensitivity of 73.53%, specificity of 97.40%, and diagnostic accuracy of 90.09%. A detailed analysis of diagnostic performance is shown in Table 3.

**Table 3:** Comparison of TOP100 detection for P2 and P1 lesions (per-patient analysis)

	Total patients with P2 lesions	Total patients with P1 lesions
Sensitivity, % (95% CI)	90.48 (82.09–95.80)	73.53 (55.64–87.12)
Specificity, % (95% CI)	100 (87.23–100)	97.40 (90.93–99.68)
PPV, % (95% CI)	100 (94.01–100)	92.59 (75.83–98.03)
NPV, % (95% CI)	77.14 (63.58–86.71)	89.29 (82.62–93.59)
Accuracy, % (95% CI)	92.79 (86.29–96.84)	90.09 (82.96–94.95)

*PPV* positive predictive value; *NPV* negative predictive value, *CI* confidence interval

### TOP100 detection performance—per-lesion analysis

Globally, 266 P2 lesions were identified by SR, of these 235 (88.35%) were detected by TOP100. In more detail, TR detected 100% of active bleeding (12/12), 91.3% of angiodysplasias (189/207), 79.3% of ulcers (23/29), 75% of multiple erosions areas (3/4) and 57.1% of varices (8/14). TR showed a total sensitivity of 88.35% (95% CI 83.87–91.94) and a specificity of 55% (95% CI 39.32–68.19) with PPV of 91.09% (95% CI 88.29–93.26) and NPV of 46.55 (95% CI 32.44–56.96). A complete analysis is shown in Table 4.

**Table 4:** TOP100 diagnostic performance – per-lesion analysis

	Sensitivity	Specificity	PPV	NPV	Accuracy
Total P2 lesions, % (95% CI)	88.35% (83.87–91.94)	55.00 (39.32–68.19)	91.09 (88.29–93.26)	46.55 (36.44–56.96)	82.91 (78.38–86.89)
Angiodysplasias, % (95% CI)	91.30 (86.61–94.76)	74.16 (63.79–82.86)	89.15 (85.22–92.13)	78.57 (69.87–85.29)	86.15 (81.68–89.87)
Active bleeding, % (95% CI)	100 (69.87–100)	100 (83.42–100)	100 (69.87–100)	100 (83.42–100)	100 (90.51–100)
Ulcers, % (95% CI)	79.31 (60.28–92.01)	98.95 (94.27–99.97)	95.83 (76.44–99.39)	94.00 (88.48–96.97)	94.35 (88.71–97.70)
Erosions areas, % (95% CI)	75.00 (21.94–98.68)	100 (83.42–100)	100 (30.99–100)	96.15 (78.41–99.79)	96.55 (82.24–99.91)
Varices, % (95% CI)	57.14 (28.86–82.34)	100 (86.28–100)	100 (59.77–100)	94.59 (90.53–96.97)	94.96 (89.35–98.13)

*PPV* positive predictive value; *NPV* negative predictive value

### Predictors of concordance between TOP100 and standard reading

TOP100 and SR showed a substantial agreement (k 0.75). In 84 patients, TR and SR were concordant as per-lesion type and the number of lesions. Potential predictors of concordance were analyzed, as shown in Table 5. At univariate analysis, the number of days from the first bleeding episode, an adequate cleansing, and intestinal transit time (ITT) were associated with positive concordance. At multivariate analysis, adequate cleansing was the only independent predictor of concordance (OR 2.909, 95% CI 1.192–7.096).

**Table 5:** Analysis of predictors of concordance between TOP100 and SR

Variables	Univariate			Multivariate	
	Concordance	No concordance	*p*	*p*	OR (95% CI)
Age, years	65.6 (54–79)	70 (61–78)	0.254		
Sex (males), *n*	49 (58.3%)	21 (77.8%)	0.069		
Inpatients, *n*	65 (77.4%)	18 (66.7%)	0.265		
Days from first bleeding, *n*	6 (2–11)	7 (5–14)	0.049	0.195	–
Days from last bleeding, *n*	1 (1–8)	5 (1–14)	0.08	0.259	–
Melena, *n*	55 (64.3%)	17 (63%)	0.901		
Hemoglobin, g/dL	6 (6.8–11.1)	7.5 (6.3–9.2)	0.079		
Cleansing (adequate), *n*	56 (66.7%)	11 (40.7%)	0.017	0.019	2.909 (1.192–7.096)
Prokinetic use, *n*	62 (73.8%)	18 (66.7%)	0.472		
ITT, minutes	262 (162–331)	344 (253–420)	0.02	0.078	–

*ITT* intestinal transit time

## Discussion

The growing complexity of endoscopic procedures and the advances in optical diagnosis claim higher precision and shorter procedure times, thus opening the door to artificial intelligence as a promising technology for a rapid and effective diagnosis. [15, 16] “Suspected Blood Indicator” was one of the first AI tools to be introduced in Pillcam® capsule endoscopy reading software, showing a very low sensitivity of 55.3% and specificity of 57.8% for bleeding or potentially bleeding lesions. [17] Current AI tools applied to capsule endoscopy have shown a sensitivity of 80 to 99% and a specificity of 94 to 99% in case of gastrointestinal hemorrhage, with discordant results depending on AI algorithm complexity and clinical setting. [18]

The present study focuses on the efficacy of the AI tool TOP100 for the diagnosis of suspected overt SBB in Pillcam® capsule endoscopy. In per-patient analysis, TR detected 90.5% of patients with significant P2 lesions identified by SR, thus showing a diagnostic accuracy of 92.79% with high specificity (100%) and PPV (100%), independent of lesion type; moreover, considering patients with active bleeding and angiodysplasias, sensitivity accounted for 97%–100% with a specificity of 100%, thus correctly identifying patients with potentially treatable lesions. In the case of P1 lesions, which present a lower bleeding potential, TOP100 showed inferior performance in per-patient analysis. It exhibited a sensitivity of 73.53%, specificity of 97.4%, and diagnostic accuracy of 90.09%. Consequently, a second reading is necessary to fully report P1 lesions.

In per-lesion analysis, TR detected 88.35% of P2 lesions diagnosed by SR. TOP100 showed the highest accuracy for intestinal bleeding (100%), whereas, in the case of angiodysplasias, it showed a sensitivity of 91.3% and a specificity of 74.16% with a total accuracy of 86.15%. Sensitivity decreased with other P2 lesions such as ulcers (79%), areas with multiple erosions (75%), and non-bleeding varices (57%).

To the best of our knowledge, this is the first comprehensive study to illustrate the diagnostic accuracy of TOP100. Present results are in line with a previous study by Ariera et al. who evaluated TOP100 in SBB, showing an 83.5% detection rate for patients with P2 lesions (91.2% for patients with angiodysplasias), although with a lower detection rate (54.5%) for P1 lesions. [10] A similar multicenter study by Saurin et al. described the detection ability for P2 and P1 lesions of another Pillcam® AI tool, Quick-view, describing a sensitivity of 85.1% and specificity of 84.7% in per-patient analysis and 89.2% and 84.7% in per-lesion analysis [19].

In the case of positive significant findings, TOP100 permits an adequate definition of lesions and presumptive location based on the Pillcam reading software detector or intestinal transit time, without the need for SR. [20] Moreover, in patients with active bleeding, which represents a round-clear urgent case, TOP100 enables a complete reliance on AI assessment. These two features allow the physician to take the appropriate therapeutic decision.

Both per-patient and per-lesion analysis show an insufficient NPV of 77.14% and 44.65%, respectively; this percentage of miss rate implies that completely negative explorations must pass a second conventional reading to finally discard the presence of significant lesions. Both present study results and previously published studies indicate that TOP100 is mainly designed for the detection of red-colored lesions, showing high sensitivity and NPV for active bleeding and angiodysplasias, probably due to the abundant red color in frames, yet lower results for ulcers and varices, in the white and blue/green color spectrum. [10] Moreover, while the use of cleansing in the human review of the SBCE video has not been associated with an increased diagnostic yield, [5] the analysis of predictors of concordance between TR and SR showed that adequate intestinal cleansing significantly increases lesion detection with TOP100 (OR 2.909, *p* = 0.019). This finding suggests that when employing AI-based reading, the use of a cleansing agent is recommended.

The study strength of the study is the description of the impact of AI on reading time. A conventional reading requires between 30 and 60 min. [7, 21, 12] A previous study by Saurin et al. demonstrated a significant time reduction with the Pillcam AI tool Quick-view with a median reading time of 11 min. [19] TOP100 shows a radical change from a median time of 23 min for SR to 1.8 min for TR with a time reduction of 91.7%. In cases of overt SBB, the medical priority is to rapidly identify the potential bleeding source and schedule the appropriate treatment. [9] The great benefit of TOP100 reading appears to be its rapidity which permits an early schedule of complementary diagnostic or therapeutic procedures.

The study’s main limitation is its retrospective design; however, two new independent readings were performed based on anonymized videos to reduce potential biases. Moreover, this study was designed to directly compare TOP100 with the standard reading as the gold standard, therefore it lacks the second review of both TOP100 and standard reading to confirm or discard possible false positive and negative cases and this might potentially underestimate its real diagnostic performance.

All in all, TOP100 showed the ability to detect 90.5% of patients with significant lesions and 100% of active bleeding with a median reading time of less than 2 min. Rapidity and high sensitivity make TOP100 an ideal tool for a quick review of SBCE videos and for prioritizing therapeutics in patients with overt SBB. A second standard reading is still required in most cases to fully exclude the presence of lesions, however, the clinical impact of this miss rate is still unknown and should be evaluated in future prospective clinical trials.
